# Emerging Roles of Tumor Necrosis Factor-Stimulated Gene-6 in the Pathophysiology and Treatment of Atherosclerosis

**DOI:** 10.3390/ijms19020465

**Published:** 2018-02-05

**Authors:** Rena Watanabe, Yuki Sato, Nana Ozawa, Yui Takahashi, Shinji Koba, Takuya Watanabe

**Affiliations:** 1 Laboratory of Cardiovascular Medicine, Tokyo University of Pharmacy and Life Sciences, 1432-1 Horinouchi, Hachioji-City, Tokyo 192-0392, Japan; s096241@toyaku.ac.jp (R.W.); s139052@toyaku.ac.jp (Y.S.); s139021@toyaku.ac.jp (N.O.); s126106@toyaku.ac.jp (Y.T.); 2 Division of Cardiology, Department of Medicine, Showa University School of Medicine, 1-5-8 Hatanodai, Shinagawa-ku, Tokyo 142-8666, Japan; skoba@med.showa-u.ac.jp

**Keywords:** TSG-6, atherosclerosis, endothelial cell, macrophage, vascular smooth muscle cell, coronary artery disease

## Abstract

Tumor necrosis factor-stimulated gene-6 (TSG-6) is a 35-kDa glycoprotein that has been shown to exert anti-inflammatory effects in experimental models of arthritis, acute myocardial infarction, and acute cerebral infarction. Several lines of evidence have shed light on the pathophysiological roles of TSG-6 in atherosclerosis. TSG-6 suppresses inflammatory responses of endothelial cells, neutrophils, and macrophages as well as macrophage foam cell formation and vascular smooth muscle cell (VSMC) migration and proliferation. Exogenous TSG-6 infusion and endogenous TSG-6 attenuation with a neutralizing antibody for four weeks retards and accelerates, respectively, the development of aortic atherosclerotic lesions in ApoE-deficient mice. TSG-6 also decreases the macrophage/VSMC ratio (a marker of plaque instability) and promotes collagen fibers in atheromatous plaques. In patients with coronary artery disease (CAD), plasma TSG-6 levels are increased and TSG-6 is abundantly expressed in the fibrous cap within coronary atheromatous plaques, indicating that TSG-6 increases to counteract the progression of atherosclerosis and stabilize the plaque. These findings indicate that endogenous TSG-6 enhancement and exogenous TSG-6 replacement treatments are expected to emerge as new lines of therapy against atherosclerosis and related CAD. Therefore, this review provides support for the clinical utility of TSG-6 in the diagnosis and treatment of atherosclerotic cardiovascular diseases.

## 1. Introduction

Atherosclerotic cardiovascular diseases, such as coronary artery disease (CAD) and stroke, are a leading cause of mortality worldwide [1]. The prevalence of traditional risk factors for CAD, such as diabetes, hypertension, dyslipidemia, obesity, and others, has been increasing worldwide, with consequent increases in the rates of coronary and cerebrovascular events [1]. Atherosclerosis is characterized by a complex interaction of vascular endothelial injury, inflammation with monocyte adhesion to endothelial cells (ECs), lipid deposition with macrophage foam cells, and the migration and proliferation of vascular smooth muscle cells (VSMCs) accompanied by extracellular matrix (ECM) remodeling [2].

We have investigated the atheroprotective properties of a large number of novel human endogenous peptides, such as salusin-α, heregulin-β1, omentin-1, catestatin-1, urocortin-1, stanniocalcin-1, and incretins (glucagon-like peptide-1, glucose-dependent insulinotropic polypeptide) [3,4,5,6,7,8,9]. Now we are paying more attention to tumor necrosis factor-stimulated gene-6 (TSG-6) as an anti-atherogenic protein [10]. There has been recent progress in TSG-6 studies. The present article reviews the recent literature and introduces our new data regarding the role of TSG-6 in the pathogenesis of atherosclerosis and as a biomarker and novel potential therapeutic target for atherosclerotic cardiovascular diseases.

## 2. Structure of TSG-6

TSG-6, which maps to human chromosome 2q23.3, was originally identified as the sixth gene product induced by tumor necrosis factor-α (TNF-α) in human fibroblasts [11], and is also called Tnfaip6 or Tnfip6 (accession numbers: CAD12353, CAD13434). TSG-6 cDNA encodes a polypeptide of 277 amino acids, including an N-terminal cleavable signal peptide of 17 amino acids. The molecular size of the mature, fully glycosylated TSG-6 protein is 31,203 Da. The N-terminal half of the TSG-6 protein shows 36–40% homology to the Link module, which is a conserved sequence in hyaluronan-binding proteins, such as CD44, cartilage link protein, and G1 domains of aggrecan and versican [12]. The C-terminal half of the molecule (CUB domain) shows 30% homology to the complement C1r A chain [12]. Human and murine TSG-6 are >94% identical [12]. The specific receptors for TSG-6 have not yet been identified.

## 3. Expression and Regulation of TSG-6

TSG-6 is not usually expressed but is rapidly upregulated in many different cell types, including monocytes/macrophages, dendritic cells, leukocytes, ECs, VSMCs, fibroblasts, synoviocytes, chondrocytes, and proximal tubular epithelial cells upon exposure to inflammatory mediators, such as interleukin (IL)-1, interferon-γ, TNF-α, lipopolysaccharide (LPS), and prostaglandin E_2_ [13,14,15] as well as growth factors, such as fibroblast growth factor, epidermal growth factor, and transforming growth factor-β, and mechanical stimuli in vitro [12,16]. Expression of TSG-6 is also known to be markedly upregulated by exposure to high glucose and fatty acid (palmitic acid) concentrations in human umbilical vein endothelial cells (HUVECs) [17]. In contrast, TSG-6 expression is suppressed by anti-inflammatory cytokines, such as IL-4 or IL-10 either directly or via inhibition of LPS/Toll-like receptor (TLR)-induced cell activation [15].

TSG-6 is released from the secretory granules of neutrophils and mast cells as well as macrophages and a wide variety of stromal cell types [12,15,18,19,20]. Mesenchymal stem cells (MSCs) also secrete TSG-6 to repair tissue injury and wounds and reduce inflammation [21]. Several lines of evidence have shown that TSG-6 is detected in synovial fluids and joint tissues from patients with rheumatoid arthritis and osteoarthritis [13,22] and in sera of patients with bacterial sepsis, systemic lupus erythematosus, and CAD [10,23].

## 4. Roles of TSG-6

Based on structural homologies, TSG-6 binds to a large number of components of the ECM including hyaluronan, heparin, heparan sulfate, thrombospondins-1 and -2, fibronectin, and pentraxin-3 [24,25]. These interactions primarily act to stabilize or remodel the ECM [25]. Several lines of evidence have suggested that TSG-6 may play a crucial role in ECM formation, inflammatory cell migration, cell proliferation, and developmental processes [26]. TSG-6 is known as a potent inhibitor of neutrophil migration and can modulate the protease network by inhibiting plasmin [24]. TSG-6 also increases hyaluronan synthesis in airway smooth muscle cells [26]. TSG-6 has a chondroprotective effect, with reduced loss of cartilage proteoglycan and less accumulation of matrix metalloproteinase (MMP) and aggecanase-generated aggrecan fragments [27]. TSG-6 is locally coexpressed with its ligand pentraxin-3 that cooperates with TSG-6 in ECM assembly [28]. TSG-6 suppresses the inhibitory effects of pentraxin-3 on fibroblast growth factor-2-mediated angiogenesis [28].

TSG-6 has been shown to exert anti-inflammatory effects in experimental models of arthritis, corneal wounding, acute myocardial infarction, and acute cerebral infarction [29,30,31,32]. In transgenic mice, inactivation of the gene increases inflammatory responses [33], and over-expression of the gene decreases inflammatory responses [34]. Administration of recombinant TSG-6 decreases LPS-induced inflammation (IL-6 and interferon-γ), and improves arthritis and memory after traumatic brain injury in several murine models [25,29,35,36]. The half-life of TSG-6 after intravenous injection into mice is up to 0.2 h [25].

In addition, TSG-6 secreted by human adipose tissue-derived MSCs ameliorates dextran sulfate sodium-induced colitis by inducing anti-inflammatory M2 macrophage polarization in mice [37]. Co-incubation of human adipose tissue-derived MSCs suppresses LPS-induced IL-1β secretion from THP-1 monocytes via increasing TSG-6 expression [38]. Knocking down TSG-6 in MSCs abrogates the inhibitory effects of MSCs on inflammatory neovascularization and monocyte/macrophage infiltration [39]. The intraarticular injection of MSCs increases TSG-6 expression in joint cartilage and inhibits monoiodoacetate-induced arthritis in rats [40].

**Figure 1:**
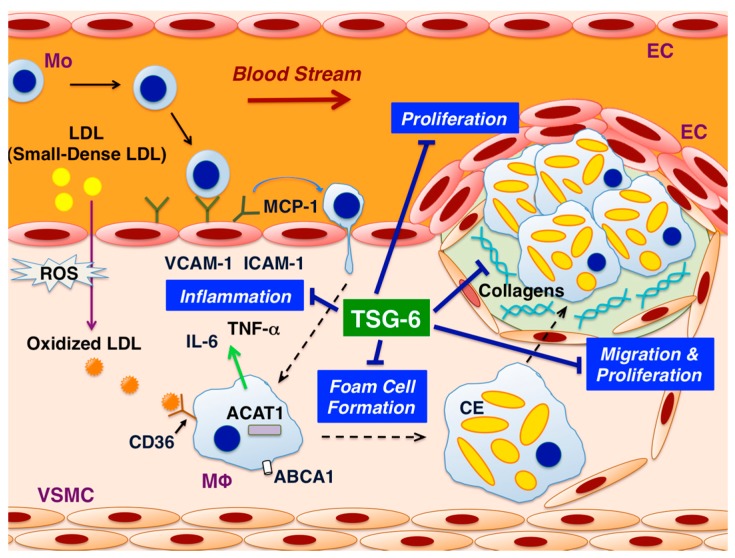
Mechanisms underlying the atheroprotective effects of tumor necrosis factor-stimulated gene-6 (TSG-6). This figure illustrates the suppressive effects of TSG-6 on atherosclerotic plaque formation in the arterial wall. TSG-6 prevents atherosclerosis by suppressing the inflammatory responses in endothelial cells (ECs) and macrophages, oxidized low-density lipoprotein (LDL)-induced foam cell formation in macrophages, and the migration and proliferation of vascular smooth muscle cells (VSMCs). TSG-6 increases the production of collagen-1 and -3 by VSMCs. Abbreviations: ACAT1 = acyl-coenzyme A:cholesterol acyltransferase-1; ABCA1 = ATP-binding cassette transporter A1; CE = cholesterol ester; ICAM-1 = intercellular adhesion molecule-1; MCP-1 = monocyte chemotactic protein-1; Mo = monocyte; MΦ = macrophage; ROS = reactive oxygen species; VCAM-1 = vascular cell adhesion molecule-1.

Several lines of evidence have recently shown the atheroprotective effects of TSG-6. TSG-6 exerts anti-atherosclerotic effects on all the three cellular players in the pathogenesis of atherosclerosis, such as ECs, macrophages, and VSMCs (Figure 1). Although the receptors for TSG-6 have not yet been clarified, it is almost certain that the receptors are present in three types of vascular cells. Next, the present review introduces the anti-atherosclerotic effects of TSG-6 on vascular cells in vitro, animal models in vivo, and human clinical data, in this order.

## 5. Effects of TSG-6 in ECs

TSG-6 inhibits leukocyte adhesion to HUVECs and HUVEC-derived EA.hy926 ECs and leukocyte migration [41,42]. TSG-6 suppresses the proliferation of EA.hy926 ECs [10]. TSG-6 suppresses LPS-induced expression of monocyte chemotactic protein-1 (MCP-1), vascular cell adhesion molecule-1, and intercellular adhesion molecule-1 in HUVECs [10]. In addition, MSCs improve glucolipotoxicity via TSG-6 production in HUVECs [17].

## 6. Effects of TSG-6 in Monocytes/Macrophages

TSG-6, either directly or through a complex with hyaluronan, binds to CD44 and downregulates TLR-2/nuclear factor-κB (NF-κB) and upregulates cyclooxygenase-2, a negative regulator of inflammation, in mouse macrophages [43,44]. TSG-6 inhibits cell proliferation and LPS-induced release of inflammatory cytokines, such as IL-1β, IL-6, and TNF-α, via c-Jun N-terminal kinase (JNK) and p38 pathways in rat macrophages [45]. TSG-6 also suppresses the inflammatory M1 phenotype and LPS-induced TNF-α production in human monocyte-derived macrophages [10]. TSG-6 suppresses oxidized low-density lipoprotein-induced foam cell formation associated with downregulation of acyl-coenzyme A:cholesterol acyltransferase-1 and CD36 in human monocyte-derived macrophages [10].

## 7. Effects of TSG-6 in VSMCs

TSG-6 suppresses angiotensin II-induced migration and proliferation of human aortic smooth muscle cells via c-Src/JNK/NF-κB pathways [10]. In contrast, TSG-6 enhances rat VSMC proliferation [46]. TSG-6 also increases the expression of collagen-1, collagen-3, MMP-2, and tissue inhibitor of metalloproteinase-2 in human aortic smooth muscle cells [10]. These findings indicate that TSG-6 suppresses atherosclerosis and restenosis after angioplasty and induces vascular remodeling.

## 8. Effects of TSG-6 on Atherosclerotic Lesion Development in ApoE-Deficient Mice

**Figure 2:**
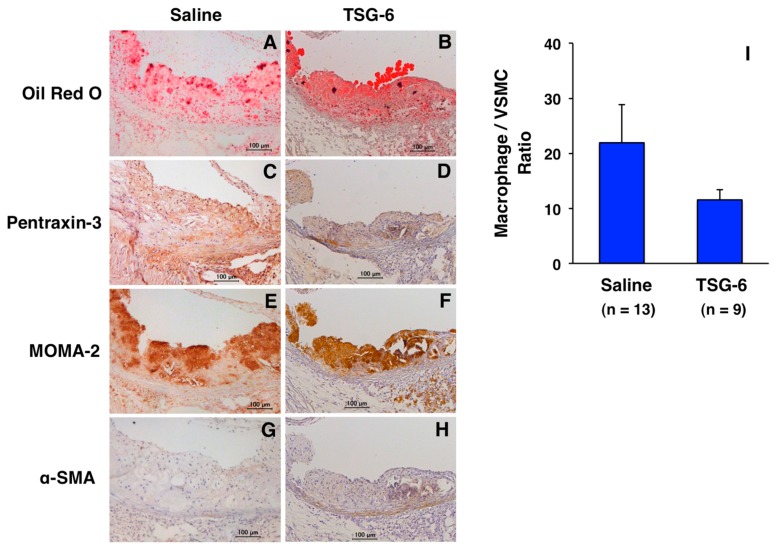
Effects of TSG-6 on atheromatous plaque progression and phenotype in ApoE-deficient mice. Four-week infusion of TSG-6 (100 μg/mouse, GenWay Biotech, San Diego, CA, USA) or saline (control) using osmotic mini-pumps was performed in 17-week-old ApoE-deficient mice fed a high cholesterol diet. Atheromatous plaques (**A**,**B**); vascular inflammation (**C**,**D**); macrophage contents (**E**,**F**); and VSMC contents (**G**,**H**) were assessed by staining with Oil Red O (Wako Pure Chemical Industries, Osaka, Japan), anti-pentraxin-3 antibody (Bioss Antibodies, Woburn, MA, USA), anti-MOMA-2 antibody (Millipore, Billerica, MA, USA), or anti-α-SMA antibody (Sigma, St. Louis, MO, USA), respectively. Hematoxylin was used for nuclear staining. Bar = 100 μm. The markers of plaque instability, such as high levels of pentraxin-3 expression and the increased ratio of macrophage contents (μm^2^)/VSMC contents (μm^2^) within atheromatous plaques (**I**), were compared between 21-week-old mice infused with saline (*n* = 13) and TSG-6 (*n* = 9). Data are presented as mean ± SEM. Unpaired Student’s *t* test was used for statistical analysis. *p* = 0.1453. These results are unpublished data from our previous experiments [10].

**Figure 3:**
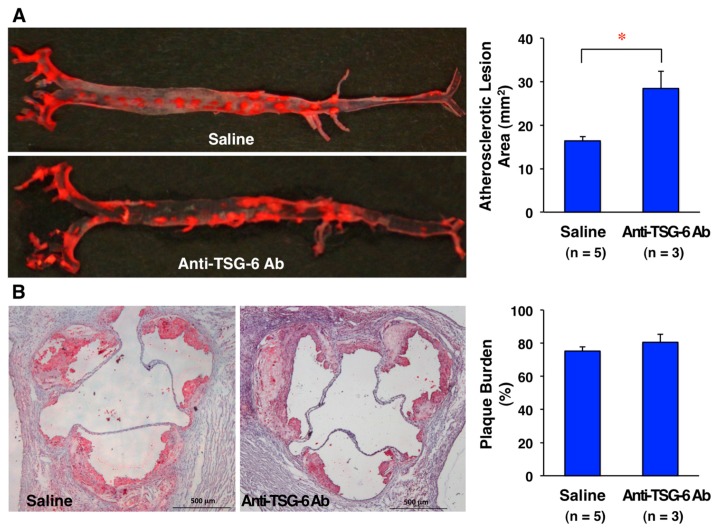
Effects of endogenous TSG-6 decreases on the development of atherosclerotic lesions in ApoE-deficient mice. Four-week infusion of an anti-TSG-6 neutralizing antibody (50 μg/mouse, R&D Systems, Minneapolis, MN, USA) or saline (control) using osmotic mini-pumps was performed in 17-week-old ApoE-deficient mice fed a high cholesterol diet. Atherosclerotic lesions were stained with Oil Red O (Wako Pure Chemical Industries, Osaka, Japan). The atherosclerotic lesions were measured in the whole aortic tree. Plaque burden is expressed as a percentage relative to the entire cross section of the aortic sinus wall. Bar = 500 μm. Atherosclerotic lesions in the entire aortic surface area (**A**) and plaque burden (**B**) are increased in mice infused with anti-TSG-6 neutralizing antibody (*n* = 3) than those infused with saline (*n* = 5). Data are presented as mean ± SEM. Unpaired Student’s *t* test was used for statistical analysis. (**A**) * *p* = 0.0085; (**B**) *p* = 0.3124. These results are unpublished data from our new experiments.

Infusing TSG-6 (100 μg/mouse) into ApoE-deficient mice for four weeks retards the development of atherosclerotic lesions in the entire surface area of the aorta [10]. In atheromatous plaques in the aortic sinus wall, vascular inflammation (pentraxin-3) and monocyte/macrophage and VSMC contents are decreased (Figure 2D,F,H) by TSG-6 infusion. The ratio of macrophage contents/VSMC contents (a surrogate marker of plaque instability) is reduced by TSG-6 infusion (Figure 2I). TSG-6 also increases collagen fibers within atheromatous plaques [10]. These findings indicate that TSG-6 stabilizes atheromatous plaques. In addition, four-week infusion of anti-TSG-6 neutralizing antibody (50 μg/mouse) into ApoE-deficient mice significantly enhances the development of atherosclerotic lesions in the entire aortic surface area and plaque burden in the aortic sinus (Figure 3A,B).

Four-week infusion of TSG-6 (100 μg/mouse) into ApoE-deficient mice decreases the inflammatory M1 phenotype and the levels of inflammasome proteins such as MCP-1, NF-κB, C-reactive protein, and apoptosis-associated speck-like protein containing a caspase recruitment domain in exudate peritoneal macrophages [10]. TSG-6 also decreases plasma levels of total cholesterol with a tendency to increase high-density lipoprotein cholesterol in ApoE-deficient mice [10].

## 9. Roles of TSG-6 in Animal Atherosclerosis and Vascular Restenosis in Wire-Injury and Vein Graft Models

**Figure 4:**
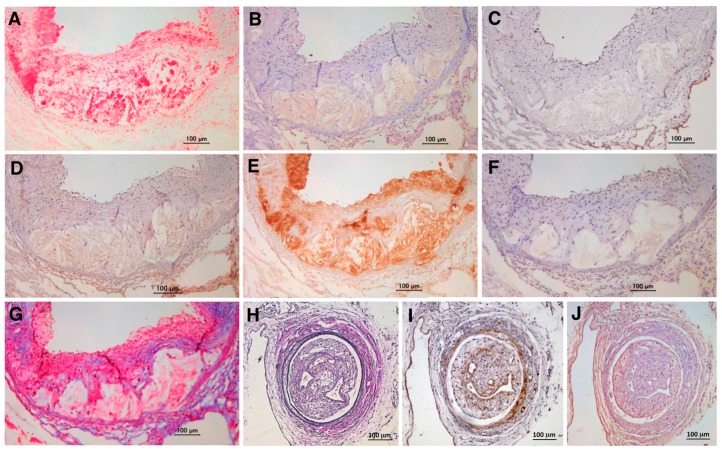
Expression of TSG-6 in atherosclerotic and restenotic lesions in ApoE-deficient mice. TSG-6 is expressed at high levels in aortic atherosclerotic lesions (**A**–**G**) and femoral artery restenotic lesions after wire injury (**H**–**J**) in ApoE-deficient mice fed a high-cholesterol diet. Tissues were immunostained with Oil Red O (Wako Pure Chemical Industries, Osaka, Japan; (**A**)); anti-TSG-6 antibody (Bioworld Technology, St. Louis Park, MN, USA; (**B**,**J**)); anti-podocalyxin antibody (Life Technologies, Carlsbad, CA, USA; (**C**)); anti-pentraxin-3 antibody (Bioss Antibodies, Woburn, MA, USA; (**D**)); anti-MOMA-2 antibody (Millipore, Billerica, MA, USA; (**E**)); anti-α-SMA antibody (Sigma, St. Louis, MO, USA; (**F**,**I**)); Masson’s Trichrome (Muto Pure Chemicals, Tokyo, Japan; (**G**)); and Elastica-Van Gieson (Muto Pure Chemicals; (**H**)). Hematoxylin was used for nuclear staining. Bar = 100 μm. (**C**) Podocalyxin is used as an EC marker. (**G**) Masson’s Trichrome is used to stain collagen fibers in blue. Panels (**A**–**G**) show unpublished data from our previous experiments [10], and panels (**H**–**J**) show unpublished data from our new experiments.

Several animal studies have shown the expression of TSG-6 in atherosclerotic lesions in vivo [10,46,47]. TSG-6 is localized in rat neointima after arterial injury and rabbit carotid atherosclerotic plaques [46,47]. In our study, TSG-6 was expressed in atherosclerotic plaques in the aorta and neointimal lesions in the femoral artery after wire injury in ApoE-deficient mice (Figure 4B,J). The expression of TSG-6 is consistent with vascular inflammation and macrophages in atheromatous plaques (Figure 4B,D,E) and neointimal thickness (VSMCs) in obstructive arteries following injury (Figure 4I,J).

TSG-6 inhibits the inflammatory response of transplanted vein grafts in rats and reduces vascular injury by downregulating the JNK and P38 pathways [45]. TSG-6 suppresses vascular restenosis in the venous bridge with decreasing VSMC proliferation, macrophage infiltration, and plasma levels of inflammatory cytokines, such as IL-1β, IL-6, and TNF-α, via JNK and p38 pathways [45].

## 10. Expression of TSG-6 in Human Arteriosclerotic Lesions and Aneurysms

Recent studies have shown the presence of TSG-6 expression in human coronary atherosclerotic plaques and abdominal aortic aneurysms [10,48]. The expression of TSG-6 is almost absent in normal and non-stenotic coronary arteries from non-CAD and CAD patients respectively [10]. In the stenotic coronary arteries from CAD patients, TSG-6 is highly expressed in the fibrous cap within atherosclerotic plaques [10]. In addition, the expression of TSG-6 is observed in the tunica media of human abdominal aortic aneurysms [48]. These findings suggest that TSG-6 is produced to counteract the progression of atherosclerotic lesions and to stabilize vulnerable plaques and aneurysms.

## 11. Potential Biomarker for CAD

**Figure 5:**
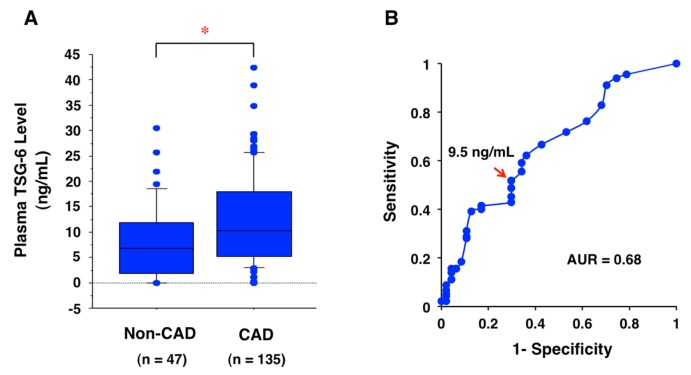
Biomarker of CAD using plasma TSG-6 levels at the onset of acute coronary syndrome. (**A**) Plasma TSG-6 levels are significantly higher in 135 CAD patients than 47 non-CAD subjects. Data are shown as median (center line in box) with 25th percentile (lower line of box), 75th percentile (upper line in box), 10th percentile (bars below box), and 90th percentile (bars above box). Unpaired Student’s *t* test was used for statistical analysis. * *p* = 0.0013. The graph was modified from the graph shown in our previous report (Watanabe et al., Atheroprotective Effects of Tumor Necrosis Factor–Stimulated Gene-6; JACC: Basic to Translational Science, **2016**, *6*, 494–509, permitted by Elsevier) [10]. (**B**) The receiver operating characteristic (ROC) curve analysis indicates the cutoff value of plasma TSG-6 for detecting CAD. The arrow shows the cutoff value (9.5 ng/mL) chosen to gain a relatively higher true-positive rate (sensitivity) with a lower false-positive rate (1-specificity). The area under the curve (AUC) value is 0.68. The results are unpublished data from our previous study [10].

Plasma TSG level was significantly higher in 135 patients with angiographically proven CAD (acute coronary syndrome) than the level in 47 non-CAD subjects [10] (Figure 5A). Based on the receiver operating characteristic (ROC) curve (Figure 5B), 9.5 ng/mL is adopted as the cutoff value that shows a higher true-positive rate (sensitivity) with a low false-positive rate (1-specificity). The area under the curve (AUC) value is 0.68 (Figure 4B). The sensitivity and specificity for detecting CAD using this cutoff value were 52% and 30%, respectively. Therefore, plasma TSG-6 level could be a reliable biomarker for detecting CAD.

**Figure 6:**
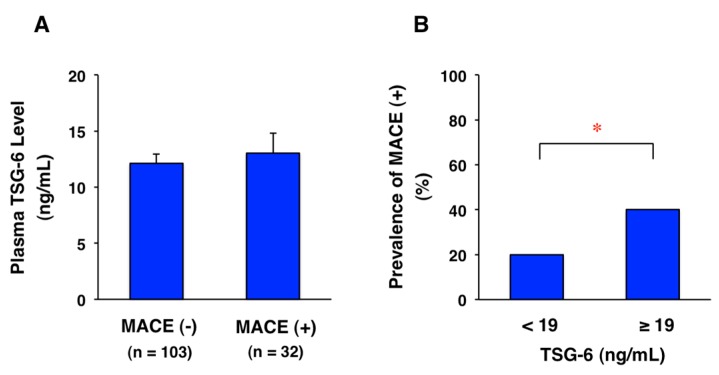
Prediction of prognosis using plasma TSG-6 levels at onset of CAD. Among 135 patients with acute coronary syndrome, 32 patients had major adverse cardiovascular events (MACE) including cardiovascular death, heart failure, acute myocardial infarction, post-infarction angina, and ischemic stroke during a period of four years after onset. The follow-up period was 50.0 ± 0.57 months. (**A**) Plasma TSG-6 levels tended to increase in CAD patients with MACE compared with those without MACE. Data are presented as mean ± SEM. Unpaired Student’s *t* test was used for statistical analysis. *p* = 0.6109; (**B**) Prevalence rate of MACE (+) was significantly higher in CAD patients with TSG-6 ≥ 19 ng/mL (40%, 10/25 cases) than those with TSG-6 < 19 ng/mL (20%, 22/110 cases). Chi-squared test was used for statistical analysis. * *p* = 0.0338. These results are unpublished data from our follow-up study based on our previous report [10].

Among 135 patients with CAD, 32 patients had major adverse cardiovascular events (MACE), defined as cardiovascular death, heart failure, acute myocardial infarction, post-infarction angina, and ischemic stroke, during a period of four years. Plasma TSG-6 level at the onset of acute coronary syndrome tended to be increased in CAD patients with MACE compared with those without MACE, but there was no significant difference (Figure 6A). Furthermore, the prevalence rate of MACE was significantly higher in CAD patients with TSG-6 ≥ 19 ng/mL (40%, 10/25 cases) than the rate in those with TSG-6 < 19 ng/mL (20%, 22/110 cases) (Figure 6B). The finding suggests that high plasma levels of TSG-6 may induce downregulation of its receptor, leading to ineffective MACE prevention in CAD patients.

## 12. Treatment of Atherosclerotic Diseases with TSG-6

There are several major therapeutic strategies against atherosclerosis and related diseases using TSG-6. One is the enhancement of endogenous TSG-6 by MSC infusion and the another is the supplementation with exogenous TSG-6. (1) Silencing TSG-6 in the administered MSCs results in loss of therapeutic activity, whereas administering exogenous TSG-6 rescues the therapeutic activity [21]. MSC therapeutic activities in other animal models of disease including cerebral ischemia, myocardial infarction, type 1 diabetes, peritoneal adhesions, and experimental autoimmune encephalomyelitis were observed to be dependent on TSG-6 [31,32,49,50,51,52]. (2) For replacement therapy of exogenous TSG-6, a great amount of recombinant human TSG-6 is needed. The recombinant TSG-6 is synthesized in *Escherichia coli*, wheat germ, insect cells, Chinese hamster ovary (CHO) cells, and mouse myeloma cells. CHO cell-derived TSG-6 has a longer half-life compared with mouse myeloma cell-derived TSG-6 in vivo [25].

It is especially important to elucidate the putative receptor for TSG-6, which provides an investigation of its agonist as a new drug target. Otherwise, it is important to clarify a biologically active fragment that has inhibitory effects against atherosclerosis among the 260 amino acids in TSG-6 protein (excluding the signal peptide), and further to develop derivatives or analogs based on the TSG-6 protein for preventing and treating atherosclerosis. Because infusion and injection are not convenient for patients, oral administration is a more suitable method. However, TSG-6 protein is digested after oral administration. Therefore, nanocapsules have received a great amount of attention as a drug delivery method [53]. Encapsulation methods are beneficial for protecting polypeptides against the digestive environment, thereby promoting absorption and delivery to target organs [53]. Due to their small size, these particles are able to pass only through the increased endothelial gaps due to injury and blood vessel damage in atherosclerotic arteries. Nanocapsules including TSG-6 analogs would be useful for oral administration.

However, manipulating plasma TSG-6 level as a potential therapeutic strategy should be considered with caution. If the putative TSG-6 receptors are downregulated in CAD patients, simply infusing TSG-6 would not be effective. Further treatments improving TSG-6 resistance should be developed in the future.

## 13. Conclusions

The above findings indicate that TSG-6 slows the development of atherosclerotic lesions by decreasing plasma total cholesterol levels, inflammatory responses of ECs and macrophages, macrophage foam cell formation, and the migration and proliferation of VSMCs. In addition, TSG-6 contributes to plaque stability by promoting collagen production by VSMCs in the fibrous cap. Thus, endogenous TSG-6 enhancement and exogenous TSG-6 replacement treatments are expected to emerge as a new line of therapy against atherosclerosis and its related CAD. The results presented here also provide insights into the potential use of TSG-6 as a biomarker for CAD. These findings may strengthen the clinical utility of TSG-6 in the diagnosis and treatment of atherosclerotic cardiovascular diseases.

## Abbreviations

CAD	Coronary artery disease
EC	Endothelial cell
ECM	Extracellular matrix
HUVEC	Human umbilical vein endothelial cell
IL	Interleukin
JNK	c-Jun N-terminal kinase
LPS	Lipopolysaccharide
MACE	Major adverse cardiovascular event
MCP-1	Monocyte chemotactic protein-1
MMP	Matrix metalloproteinase
MSC	Mesenchymal stem cell
NF-κB	Nuclear factor-κB
TLR	Toll-like receptor
TNF-α	Tumor necrosis factor-α
TSG-6	Tumor necrosis factor-stimulated gene-6
VSMC	Vascular smooth muscle cell

## References

[1] Barquera S., Pedroza-Tobías A., Medina C., Hernández-Barrera L., Bibbins-Domingo K., Lozano R., Moran A.E. (2015). Global overview of the epidemiology of atherosclerotic cardiovascular disease. Arch. Med. Res..

[2] Hansson G.K., Libby P. (2006). The immune response in atherosclerosis: A double-edged sword. Nat. Rev. Immunol..

[3] Watanabe T., Nishio K., Kanome T., Matsuyama T., Koba S., Sakai T., Sato K., Hongo S., Nose K., Ota H. (2008). Impact of salusin-α and -β on human macrophage foam cell formation and coronary atherosclerosis. Circulation.

[4] Xu G., Watanabe T., Iso Y., Koba S., Sakai T., Nagashima M., Arita S., Hongo S., Ota H., Kobayashi Y. (2009). Preventive effects of heregulin-β_1_ on macrophage foam cell formation and atherosclerosis. Circ. Res..

[5] Watanabe K., Watanabe R., Konii H., Shirai R., Sato K., Matsuyama T., Ishibashi-Ueda H., Koba S., Kobayashi Y., Hirano T. (2016). Counteractive effects of omentin-1 against atherogenesis. Cardiovasc. Res..

[6] Kojima M., Ozawa N., Mori Y., Takahashi Y., Watanabe-Kominato K., Shirai R., Watanabe R., Sato K., Matsuyama T., Ishibashi-Ueda H. (2018). Catestatin prevents macrophage-driven atherosclerosis but not arterial injury-induced neointimal hyperplasia. Thromb. Haemost..

[7] Hasegawa A., Sato K., Shirai R., Watanabe R., Yamamoto K., Watanabe K., Nohtomi K., Hirano T., Watanabe T. (2014). Vasoprotective effects of urocortin 1 against atherosclerosis in vitro and in vivo. PLoS ONE.

[8] Yamamoto K., Tajima Y., Hasegawa A., Takahashi Y., Kojima M., Watanabe R., Sato K., Shichiri M., Watanabe T. (2016). Contrasting effects of stanniocalcin-related polypeptides on macrophage foam cell formation and vascular smooth muscle cell migration. Peptides.

[9] Nagashima M., Watanabe T., Terasaki M., Tomoyasu M., Nohtomi K., Kim-Kaneyama J., Miyazaki A., Hirano T. (2011). Native incretins prevent the development of atherosclerotic lesions in apolipoprotein E knockout mice. Diabetologia.

[10] Watanabe R., Watanabe H., Takahashi Y., Kojima M., Konii H., Watanabe K., Shirai R., Sato K., Matsuyama T., Ishibashi-Ueda H. (2016). Atheroprotective effects of tumor necrosis factor-stimulated gene-6. JACC Basic Transl. Sci..

[11] Lee T.H., Lee G.W., Ziff E.B., Vilcek J. (1990). Isolation and characterization of eight tumor necrosis factor-induced gene sequences from human fibroblasts. Mol. Cell. Biol..

[12] Milner C.M., Day A.J. (2003). TSG-6: A multifunctional protein associated with inflammation. J. Cell Sci..

[13] Wisniewski H.G., Maier R., Lotz M., Lee S., Klampfer L., Lee T.H., Vilcek J. (1993). TSG-6: A TNF-, IL-1-, and LPS-inducible secreted glycoprotein associated with arthritis. J. Immunol..

[14] Fujimoto T., Savani R.C., Watari M., Day A.J., Strauss J.F. (2002). Induction of the hyaluronic acid-binding protein, tumor necrosis factor-stimulated gene-6, in cervical smooth muscle cells by tumor necrosis factor-α and prostaglandin E_2_. Am. J. Pathol..

[15] Maina V., Cotena A., Doni A., Nebuloni M., Pasqualini F., Milner C.M., Day A.J., Mantovani A., Garlanda C. (2009). Coregulation in human leukocytes of the long pentraxin PTX3 and TSG-6. J. Leukoc. Biol..

[16] Lee R.T., Yamamoto C., Feng Y., Potter-Perigo S., Briggs W.H., Landschulz K.T., Turi T.G., Thompson J.F., Libby P., Wight T.N. (2001). Mechanical strain induces specific changes in the synthesis and organization of proteoglycans by vascular smooth muscle cells. J. Biol. Chem..

[17] An X., Li L., Chen Y., Luo A., Ni Z., Liu J., Yuan Y., Shi M., Chen B., Long D. (2016). Mesenchymal stem cells ameliorated glucolipotoxicity in HUVECs through TSG-6. Int. J. Mol. Sci..

[18] Nagyeri G., Radacs M., Ghassemi-Nejad T., Tryniszewska B., Olasz K., Hutas G., Gyorfy Z., Hacall V.C., Glant T., Mikecz K. (2011). TSG-6 protein, a negative regulator of inflammatory arthritis, forms a ternary complex with murine mast cell tryptases and heparin. J. Boil. Chem..

[19] Lee T.H., Wisniewski H.G., Vilcek J. (1992). A novel secretory tumor necrosis factor-inducible protein (TSG-6) is a member of the family of hyaluronate binding proteins, closely related to the adhesion receptor CD44. J. Cell Biol..

[20] Chang M., Chan C.K., Braun K.R., Green P.S., O’Brien K.D., Chait A., Day A.J., Wight T.N. (2012). Monocyte-to-macrophage differentiation: Synthesis and secretion of a complex extracellular matrix. J. Biol. Chem..

[21] Madrigal M., Rao K.S., Riordan N.H. (2014). A review of therapeutic effects of mesenchymal stem cell secretions and induction of secretory modification by different culture methods. J. Transl. Med..

[22] Bayliss M.T., Howat S.L., Dudhia J., Murphy J.M., Barry F.P., Edwards J.C., Day A.J. (2001). Up-regulation and differential expression of the hyaluronan-binding protein TSG-6 in cartilage and synovium in rheumatoid arthritis and osteoarthritis. Osteoarthr. Cartil..

[23] Wisniewski H.G., Vilcek J. (1997). TSG-6: An IL-1/TNF-inducible protein with anti-inflammatory activity. Cytokine Growth Factor Rev..

[24] Milner C.M., Higman V.A., Day A.J. (2006). TSG-6: A pluripotent inflammatory mediator?. Biochem. Soc. Trans..

[25] Kim D.K., Choi H., Nishida H., Oh J.Y., Gregory C., Lee R.H., Yu J.M., Watanabe J., An S.Y., Bartosh T.J. (2016). Scalable production of a multifunctional protein (TSG-6) that aggregates with itself and the CHO cells that synthesize it. PLoS ONE.

[26] Lauer M.E., Cheng G., Swaidani S., Aronica M.A., Weigel P.H., Hascall V.C. (2013). Tumor necrosis factor-stimulated gene-6 (TSG-6) amplifies hyaluronan synthesis by airway smooth muscle cells. J. Biol. Chem..

[27] Mahoney D.J., Mulloy B., Forster M.J., Blundell C.D., Fries E., Milner C.M., Day A.J. (2005). Characterization of the interaction between tumor necrosis factor-stimulated gene-6 and heparin: Implications for the inhibition of plasmin in extracellular matrix microenvironments. J. Biol. Chem..

[28] Leali D., Inforzato A., Ronca R., Bianchi R., Belleri M., Coltrini D., Di Salle E., Sironi M., Norata G.D., Bottazzi B. (2012). Long pentraxin 3/tumor necrosis factor-stimulated gene-6 interaction: A biological rheostat for fibroblast growth factor 2-mediated angiogenesis. Arterioscler. Thromb. Vasc. Biol..

[29] Bardos T., Kamath R.V., Mikecz K., Glant T.T. (2001). Anti-inflammatory and chondroprotective effect of TGF-6 (tumor necrosis factor-α stimulated gene-6) in murine models of experimental arthritis. Am. J. Pathol..

[30] Oh J.Y., Roddy G.W., Choi H., Lee R.H., Ylöstalo J.H., Rosa R.H., Prockop D.J. (2010). Anti-inflammatory protein TSG-6 reduces inflammatory damage to the cornea following chemical and mechanical injury. Proc. Natl. Acad. Sci. USA.

[31] Lee R.H., Pulin A.A., Seo M.J., Kota D.J., Ylostalo J., Larson B.L., Semprun-Prieto L., Delafontaine P., Prockop D.J. (2009). Intravenous hMSCs improve myocardial infarction in mice because cells embolized in lung are activated to secrete the anti-inflammatory protein TSG-6. Cell Stem Cell.

[32] Lin Q.M., Zhao S., Zhou L.L., Fang X.S., Fu Y., Huang Z.T. (2013). Mesenchymal stem cells transplantation suppresses inflammatory responses in global cerebral ischemia: Contribution of TNF-α-induced protein 6. Acta Pharmacol. Sin..

[33] Szántó S., Bárdos T., Gál I., Glant T.T., Mikecz K. (2004). Enhanced neutrophil extravasation and rapid progression of proteoglycan-induced arthritis in TSG-6-knockout mice. Arthritis Rheum..

[34] Mindrescu C., Dias A.A., Olszewski R.J., Klein M.J., Reis L.F., Wisniewski H.G. (2002). Reduced susceptibility to collagen-induced arthritis in DBA/1J mice expressing the TSG-6 transgene. Arthritis Rheum..

[35] Mindrescu C., Thorbecke G.J., Klein M.J., Vilcek J., Wisniewski H.G. (2000). Amelioration of collagen-induced arthritis in DBA/1J mice by recombinant TSG-6, a tumor necrosis factor/interleukin-1-inducible protein. Arthritis Rheum..

[36] Watanabe J., Shetty A.K., Hattiangady B., Kim D.K., Foraker J.E., Nishida H., Prockop D.J. (2013). Administration of TSG-6 improves memory after traumatic brain injury in mice. Neurobiol. Dis..

[37] Song W.J., Li Q., Ryu M.O., Ahn J.O., Bhang D.H., Jung Y.C., Youn H.Y. (2017). TSG-6 secreted by human adipose tissue-derived mesenchymal stem cells ameliorates DSS-induced colitis by inducing M2 macrophage polarization in mice. Sci. Rep..

[38] Carelli S., Colli M., Vinci V., Caviggioli F., Klinger M., Gorio A. (2018). Mechanical activation of adipose tissue and derived mesenchymal stem cells: Novel anti-inflammatory properties. Int. J. Mol. Sci..

[39] Song H.B., Park S.Y., Ko J.H., Park J.W., Yoon C.H., Kim D.H., Kim J.H., Kim M.K., Lee R.H., Prockop D.J. (2018). Mesenchymal stromal cells inhibit inflammatory lymphangiogenesis in the cornea by suppressing macrophage in a TSG-6-dependent manner. Mol. Ther..

[40] Ichiseki T., Shimazaki M., Ueda Y., Ueda S., Tsuchiya M., Souma D., Kaneuji A., Kawahara N. (2018). Intraarticularly-injected mesenchymal stem cells stimulate anti-inflammatory molecules and inhibit pain related protein and chondrolytic enzymes in a monoiodoacetate-induced rat arthritis model. Int. J. Mol. Sci..

[41] Cao T.V., La M., Getting S.J., Day A.J., Perretti M. (2004). Inhibitory effects of TSG-6 link module on leukocyte-endothelial cell interactions in vitro and in vivo. Microcirculation.

[42] Dyer D.P., Thomson J.M., Hermant A., Jowitt T.A., Handel T.M., Proudfoot A.E., Day A.J., Milner C.M. (2014). TSG-6 inhibits neutrophil migration via direct interaction with the chemokine CXCL8. J. Immunol..

[43] Choi H., Lee R.H., Bazhanov N., Oh J.Y., Prockop D.J. (2011). Anti-inflammatory protein TSG-6 secreted by activated MSCs attenuates zymosan-induced mouse peritonitis by decreasing TLR2/NF-κB signaling in resident macrophages. Blood.

[44] Mindrescu C., Le J., Wisniewski H.G., Vilcek J. (2005). Up-regulation of cyclooxygenase-2 expression by TSG-6 protein in macrophage cell line. Biochem. Biophys. Res. Commun..

[45] Zhang C., Zhang B., Wang H., Tao Q., Ge S., Zhai Z. (2017). Tumor necrosis factor alpha-stimulated gene-6 (TSG-6) inhibits the inflammatory response by inhibiting the activation of P38 and JNK signaling pathway and decreases the restenosis of vein grafts in rats. Heart Vessels.

[46] Ye L., Mora R., Akhayani N., Haudenschild C.C., Liau G. (1997). Growth factor and cytokine-regulated hyaluronan-binding protein TSG-6 is localized to the injury-induced rat neointima and confers enhanced growth in vascular smooth muscle cells. Circ. Res..

[47] Wang S.S., Hu S.W., Zhang Q.H., Xia A.X., Jiang Z.X., Chen X.M. (2015). Mesenchymal stem cells stabilize atherosclerotic vulnerable plaque by anti-inflammatory properties. PLoS ONE.

[48] Wang S.K., Xie J., Green L.A., McCready R.A., Motaganahalli R.L., Fajardo A., Babbey C.C., Murphy M.P. (2017). TSG-6 is highly expressed in human abdominal aortic aneurysms. J. Surg. Res..

[49] Kota D.J., Wiggins L.L., Yoon N., Lee R.H. (2013). TSG-6 produced by hMSCs delays the onset of autoimmune diabetes by suppressing Th1 development and enhancing tolerogenicity. Diabetes.

[50] Wang N., Shao Y., Mei Y., Zhang L., Li Q., Li D., Chen X. (2012). Novel mechanism for mesenchymal stem cells in attenuating peritoneal adhesion: Accumulating in the lung and secreting tumor necrosis factor α-stimulating gene-6. Stem Cell Res. Ther..

[51] Wang N., Li Q., Zhang L., Lin H., Hu J., Li D., Chen X. (2012). Mesenchymal stem cells attenuate peritoneal injury through secretion of TSG-6. PLoS ONE.

[52] Fisher-Shoval Y., Barhum Y., Sadan O., Yust-Katz S., Ben-Zur T., Lev N., Offen D. (2012). Transplantation of placenta-derived mesenchymal stem cells in the EAE mouse model of MS. J. Mol. Neurosci..

[53] Niu Z., Conejos-Sánchez I., Griffin B.T., O’Driscoll C.M., Alonso M.J. (2016). Lipid-based nanocarriers for oral peptide delivery. Adv. Drug Deliv. Rev..

